# Retraction: Cytomegalovirus and Herpes Simplex Virus Effect on the Prognosis of Mechanically Ventilated Patients Suspected to Have Ventilator-Associated Pneumonia

**DOI:** 10.1371/journal.pone.0312324

**Published:** 2024-10-14

**Authors:** The PLOS ONE Editors

The *PLOS ONE* Editors retract this article [1, 2] due to concerns about compliance with the PLOS Human Subjects Research policy.

Specifically, there are discrepancies between the ethics approval document and the reported study which were not resolved in follow-up discussions with the authors and/or institution. The article [1] states that “the study was approved by the Local Ethics Committee of the Université de la Méditerranée (Marseille, France; approval n° 07–026) which waived the need for written consent”. However, contrary to this statement the document for approval n° 07–026 states that patient consent was obtained. (Ethics approval number n° 07–026 has also been cited in [3, 4, 6]; Expressions of Concern were previously issued for two of these articles [5, 7].)

Furthermore, this article [1] does not contain sufficient information to clarify when sample collection took place and whether any samples were collected specifically for this study. The last author (LP) noted that the study used routinely performed samples and diagnostic methods.

A representative from the Aix-Marseille Université stated that the institutional investigation into the ethics concerns concluded this article meets ethical standards. They commented that the authors wrongly describe this study a prospective study and commented that this is a retrospective study instead. Furthermore, the institute’s representative stated that this study did not involve the human person and as such it did not require approval from a Comité de Protection des Personnes according to French law, and they state that there are no written consents. The institute’s statement that no consent was obtained for this study directly contradicts the statement in the IFR48 #07–026 approval document, which states that patient consent was obtained. The institute’s response did not resolve the concerns regarding the discrepancy between the information in the article and the information in the ethics approval documentation.

In addition, PLOS identified potential competing interests between the approving ethics committee and one or more of the article’s authors.

YC, SB, JMF, SH, BL, AR, CZ, MM, SJ, DR, and LP either did not respond directly or could not be reached.
